# Genetic and Epigenetic Mechanisms Underlying Phenotypic Discordance in Monochorionic Monozygotic Twins: A Systematic Review

**DOI:** 10.3390/genes17070832

**Published:** 2026-07-21

**Authors:** Dario Colacurci, Giuseppe Maria Maruotti, Gabriele Saccone, Anna Maria D’Agostino, Maria Virginia De Santis, Mariagrazia Riccardi, Mirko Martirani, Maurizio Guida, Laura Sarno

**Affiliations:** 1 Department of Public Health, University of Naples Federico II, Via Pansini 5, 80131 Naples, Italy; giuseppemaria.maruotti@unina.it (G.M.M.); anna.dagostino2@gmail.com (A.M.D.); m.virginiadesantis@gmail.com (M.V.D.S.); riccardimariagrazia99@gmail.com (M.R.); mirkoutos@gmail.com (M.M.); 2 Department of Neurosciences, Reproductive Science and Dentistry, University of Naples Federico II, Via Pansini 5, 80131 Naples, Italy; gabriele.saccone.1990@gmail.com (G.S.); maurizio.guida@unina.it (M.G.);

**Keywords:** monochorionic twins, monozygotic twins, phenotypic discordance, mosaicism, genomic sequencing, cytogenetics, epigenetics

## Abstract

Background: Monochorionic twin pregnancies provide a unique model to investigate fetal phenotypic discordance, because both fetuses share a single placenta and interconnected vascular circulation. Although most monochorionic twins are monozygotic, clinically relevant differences may arise through genetic, epigenetic, placental, and stochastic developmental mechanisms. Methods: This systematic review was conducted according to PRISMA 2020 and registered in PROSPERO (CRD420261432361). PubMed/MEDLINE, Embase, and Scopus were searched from inception to June 2026. Eligible studies included monochorionic monozygotic twin pairs with discordant congenital, developmental, or syndromic phenotypes, confirmed or clearly inferable monochorionicity, and at least one genomic, cytogenetic, or epigenetic investigation; studies describing confirmed monochorionic dizygotic twinning were excluded. Findings were synthesized qualitatively. Results: The search identified 1357 records. After duplicate removal and screening, 48 studies fulfilled the eligibility criteria, comprising 441 monozygotic twin pairs; 37 were single-pair case reports, whereas one large retrospective cohort study alone contributed 193 pairs (44% of the entire pooled sample). Reported phenotypes included congenital heart disease, chromosomal abnormalities, disorders of sex development, imprinting disorders, neurodevelopmental disease, endocrine disorders, renal anomalies, skeletal disorders, and multisystem malformations. Molecular methods included karyotyping, FISH, chromosomal microarray, array-CGH, CNV analysis, WES, WGS, targeted sequencing, and methylation profiling. Proposed mechanisms included postzygotic chromosomal errors, somatic mutations, tissue-specific mosaicism, discordant or shared CNVs, differential methylation, imprinting defects, variable expressivity, blood chimerism, unequal placental sharing, TTTS, TAPS, sFGR, and uteroplacental insufficiency. Conclusions: Phenotypic discordance in monochorionic twins is rarely explained by a single mechanism. Available evidence supports a multifactorial model in which postzygotic genetic events, epigenetic regulation, placental vascular factors, and stochastic developmental processes interact.

## 1. Introduction

Monochorionic twin pregnancies are unique in human development because both fetuses share a single placenta and an interconnected vascular circulation [1]. Although the majority of monochorionic twins are monozygotic and therefore originate from the same fertilized oocyte, important differences in structural, functional, and developmental outcomes may arise during fetal life [2]. These discordant phenotypes provide a unique opportunity to investigate the biological mechanisms responsible for human developmental variability beyond inherited genomic sequence alone [3]. Phenotypic discordance in monochorionic twins includes a broad spectrum of conditions, including chromosomal abnormalities, congenital heart disease, disorders of sex development, imprinting disorders, neurodevelopmental diseases, endocrine disorders, renal and skeletal anomalies, and multisystem congenital malformations [4,5,6,7]. In many cases, one twin is severely affected, whereas the co-twin shows no prenatally identified anomalies, despite their common placental environment and presumed genetic identity. Such observations challenge the traditional assumption that shared genotype necessarily predicts similar phenotypic outcomes and suggest that additional molecular and developmental mechanisms contribute to disease expression [8]. The pathogenesis of discordant phenotypes in monochorionic twins is likely multifactorial. Postzygotic chromosomal abnormalities, somatic mutations, tissue-specific mosaicism, differential epigenetic regulation, and variable expressivity of shared pathogenic variants have all been implicated [9,10,11,12]. At the same time, the monochorionic placenta itself may influence fetal development through unequal placental sharing and intertwin vascular anastomoses, which can in turn result in clinical sequelae such as twin-to-twin transfusion syndrome (TTTS), twin anemia-polycythemia sequence (TAPS), selective fetal growth restriction (sFGR), blood chimerism, and uteroplacental insufficiency [13]. Rather than representing competing hypotheses, genetic, epigenetic, placental, and stochastic developmental factors are increasingly recognized as interacting processes that collectively determine phenotypic variability. Recent advances in molecular diagnostics have substantially improved the investigation of discordant monochorionic twin pregnancies. Conventional cytogenetics has progressively been complemented by chromosomal microarray analysis (CMA), fluorescence in situ hybridization (FISH), whole-exome sequencing (WES), whole-genome sequencing (WGS), copy number variation (CNV) analysis, DNA methylation profiling, and other multi-omics approaches. These technologies have identified numerous postzygotic genomic and epigenomic alterations while simultaneously demonstrating that many discordant twin pairs remain genetically indistinguishable using current sequencing methods, highlighting the contribution of epigenetic regulation, tissue-specific mosaicism, and non-coding or developmental mechanisms. Nevertheless, recent literature is still heterogeneous: the majority of studies focusing on isolated case reports or small observational cohorts. Thus, a comprehensive synthesis of the available evidence is lacking. The aim of this systematic review was to summarize the molecular findings reported in monochorionic twin pregnancies with discordant phenotypes, characterize the methodologies used to investigate these cases, and critically evaluate the biological mechanisms proposed to explain phenotypic discordance.

## 2. Materials and Methods

### 2.1. Study Design and Reporting Standards

This systematic review was conducted in accordance with the Preferred Reporting Items for Systematic Reviews and Meta-Analyses (PRISMA 2020) [14] statement and the recommendations of the Cochrane Handbook for Systematic Reviews of Interventions. The review protocol was prospectively registered in the International Prospective Register of Systematic Reviews (PROSPERO) [15] (registration number CRD420261432361).

### 2.2. Literature Search

A comprehensive electronic search was performed in PubMed/MEDLINE, Embase, and Scopus from database inception through June 2026. No restrictions regarding publication date or language were applied. The complete search strategies for each database are reported in Supplementary Table S1. The search strategy combined controlled vocabulary (when available) and free-text terms related to monochorionic or monozygotic twins, phenotypic discordance, congenital abnormalities, and genomic or cytogenetic investigations, including sequencing technologies, chromosomal analyses, copy number variation, mosaicism, epigenetic profiling, and methylation studies. After completion of the electronic search, duplicate records were removed. In addition, the reference lists of all eligible articles and relevant review papers were manually screened to identify further potentially eligible studies not retrieved through database searching. The review question was structured according to the PICO framework: Population: monozygotic and/or monochorionic twin pregnancies with discordant congenital, developmental, or syndromic phenotypes; Intervention/Exposure: genomic, cytogenetic, or epigenetic investigations, including karyotyping, CMA, FISH, array comparative genomic hybridization (array-CGH), WES, WGS, targeted sequencing, methylation analysis, or other molecular genetic techniques; Comparator: the unaffected co-twin or alternative molecular findings when available; Outcomes: identification of genetic, chromosomal, or epigenetic differences potentially explaining phenotypic discordance.

### 2.3. Eligibility Criteria and Study Selection

Titles and abstracts were independently screened by two reviewers (D.C. and M.R.). Potentially relevant articles subsequently underwent full-text evaluation. Any disagreements were resolved by discussion, with arbitration by a third reviewer (L.S.) when consensus could not be achieved. Studies were considered eligible if they met all of the following criteria: (i) included monozygotic or monochorionic twin pairs with discordant phenotypes; (ii) reported monochorionicity, either prenatally, pathologically, or clearly inferable from the study; (iii) investigated the twins using at least one genomic, cytogenetic, or epigenetic technique; (iv) were original studies involving human subjects. Reviews, editorials, conference abstracts without sufficient data, animal studies, and articles lacking original molecular or cytogenetic findings were excluded. Likewise, studies in which chorionicity was not reported or could not be established were excluded. When multiple publications described the same patients, only the most informative report was retained.

### 2.4. Data Extraction

Two investigators (D.C. and M.R.) independently extracted data using a predefined standardized data collection form. Extracted variables included study characteristics (first author, publication year, country, journal, and study design), number of twin pairs, phenotypic presentation, chorionicity, method used to confirm monozygosity, molecular and cytogenetic techniques employed, tissue analyzed, principal genetic or epigenetic findings, pathogenic variants identified, implicated genes, variant classification, presumed mechanism of origin (inherited, de novo, postzygotic, or somatic), validation methods, authors’ interpretation, and overall study conclusions. Any discrepancies between reviewers were resolved through discussion until agreement was reached.

### 2.5. Quality Assessment

Methodological quality was assessed using study design-specific standardized tools. Case reports and case series were appraised using the Joanna Briggs Institute (JBI) Critical Appraisal Checklists [16]. The JBI checklist for case reports comprises eight items evaluating the clarity of patient demographics, clinical history, diagnostic methods, intervention, post-intervention outcomes, adverse events, and the educational value of the report. The JBI checklist for case series consists of ten items assessing inclusion criteria, standardized condition measurement, valid identification methods, consecutive and complete case inclusion, reporting of demographics and clinical information, outcome assessment, follow-up, statistical analysis, and overall methodological quality. Each item was rated as “Yes”, “No”, “Unclear”, or “Not applicable”, and the total score was reported as the number of criteria fulfilled. Observational studies were assessed using the Newcastle–Ottawa Scale (NOS) [17], which evaluates methodological quality across three domains: Selection (maximum 4 stars), Comparability (maximum 2 stars), and Outcome or Exposure assessment (maximum 3 stars, depending on study design), for a maximum score of nine stars. Higher scores indicate better methodological quality and lower risk of bias.

### 2.6. Data Synthesis

Considering the marked heterogeneity of the included studies with respect to clinical presentation, molecular techniques, tissues analyzed, and reported outcomes, statistical pooling was not feasible. Therefore, findings were synthesized qualitatively. The results were summarized according to study characteristics, type of discordant phenotype, molecular methodology, tissue analyzed, genetic or epigenetic alterations identified, proposed pathogenic mechanisms, and authors’ interpretation of genotype–phenotype discordance. Particular attention was given to postzygotic mutational events, chromosomal mosaicism, epigenetic differences, and tissue-specific genetic variation as potential explanations for phenotypic discordance in monochorionic monozygotic twins.

## 3. Results

### 3.1. Study Selection

**Figure 1:**
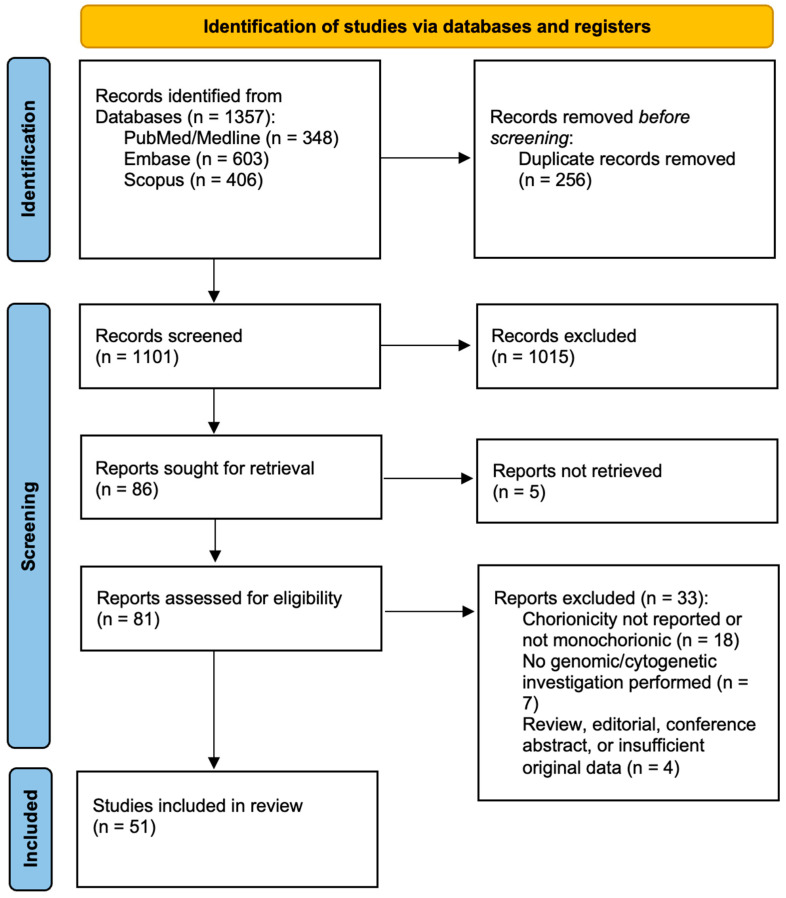
PRISMA 2020 flow diagram of the study selection process.

The literature search identified 1357 records across PubMed/MEDLINE (n = 348), Embase (n = 603), and Scopus (n = 406). After removal of 256 duplicate records, 1101 titles and abstracts were screened. Of these, 1015 records were excluded because they were not relevant to the review (e.g., wrong population, absence of monochorionic/monozygotic twins with discordant phenotypes, or no original genomic, cytogenetic, or epigenetic data reported), and 86 full-text articles were assessed for eligibility. Five reports could not be retrieved, leaving 81 articles for full-text evaluation. Thirty-three studies were subsequently excluded, mainly because chorionicity was not reported or pregnancies were not monochorionic, no genomic or cytogenetic investigation had been performed, or insufficient original data were available. Ultimately, 48 studies fulfilled the eligibility criteria and were included in the qualitative synthesis (Figure 1).

### 3.2. Methodological Quality Assessment

The methodological quality assessment is presented in Table 1. Because most included publications were case reports or small case series, the Joanna Briggs Institute (JBI) Critical Appraisal Checklists were used for these study designs, whereas observational and cohort studies were evaluated using the Newcastle–Ottawa Scale (NOS). Overall, methodological quality was moderate to high. Most case reports fulfilled 6–8 of the applicable JBI criteria, with many achieving the maximum score of 8/8. The two included case series scored 7/10 and 8/10, respectively. Observational studies generally demonstrated good methodological quality, with NOS scores ranging from 6 to 8 out of a maximum of 9 stars. The most common limitations included incomplete reporting of long-term follow-up, limited assessment of potential confounding factors in observational studies, and variability in the extent of molecular characterization. Nevertheless, confirmation of monozygosity and the application of appropriate cytogenetic or molecular investigations were consistently reported in the majority of included studies.

### 3.3. Study Characteristics

**Figure 2:**
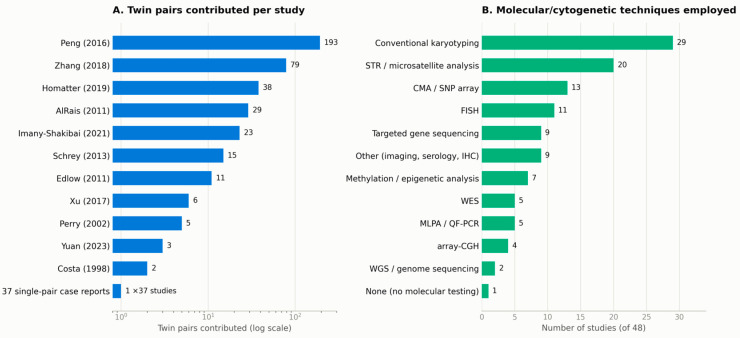
Distribution of twin pairs and molecular techniques across included studies. (**A**) Number of twin pairs contributed by each of the 11 studies reporting more than one twin pair, plotted on a logarithmic scale, alongside an aggregated bar representing the 37 studies contributing a single twin pair (case reports) [12,18,20,27,29,31,36,42,43,53,56]. (**B**) Number of included studies (of 48) employing each molecular or cytogenetic technique category; categories are not mutually exclusive, as most studies employed more than one technique.

The studies were published between 1975 and 2026 and comprised case reports, case series, retrospective cohort studies, retrospective database studies, and observational cohort studies. Overall, the review included data from 441 monozygotic twin pairs, with most studies involving monochorionic diamniotic (MCDA) pregnancies and a smaller number of monochorionic monoamniotic (MCMA) pregnancies. Studies were conducted across 16 countries, including Australia, Belgium, Brazil, Canada, China, France, Germany, Italy, Japan, the Netherlands, New Zealand, Portugal, Switzerland, Taiwan, Thailand, and the United States, with the United States, China, and Japan the most represented. The number of twin pairs per study ranged from 1, in the majority of case reports, to 193 [36]. The largest contributions came from retrospective cohort and observational studies, including Peng (2016) [36] (193 MCDA pairs), Zhang (2018) [29] (79 MCDA pairs), Homatter (2019) [27] (38 discordant MC pairs), Alrais (2011) [43] (29 MC pairs discordant for CHD), Imany-Shakibai (2021) [20] (23 MC pairs, 18 discordant), Edlow (2011) [42] (11 MCDA pairs), and Schrey (2013) [12] (10 discordant and 5 concordant MC pairs), whereas the remaining publications were case reports or small case series contributing between 1 and 6 twin pairs each. The exact number of twin pairs reported by each study is provided in Table 2. The distribution of twin pairs across studies is illustrated in Figure 2A. The most frequently investigated discordant phenotypes were congenital heart disease, chromosomal abnormalities and aneuploidies, disorders of sex development and sex chromosome mosaicism, imprinting disorders, neurodevelopmental disorders, multisystem congenital malformations, renal disorders, growth disorders, skeletal dysplasias, neurocutaneous syndromes, and endocrine disorders. Congenital heart disease represented the most commonly investigated organ system. Molecular investigations were highly heterogeneous and included conventional karyotyping, FISH, short tandem repeat (STR) analysis, CMA, array-CGH, CNV analysis, WES, WGS, targeted sequencing, DNA methylation analyses, reduced representation bisulfite sequencing (RRBS), and multi-omics approaches. The relative frequency of each technique category across the included studies is shown in Figure 2B. Biological specimens included peripheral blood, cord blood, amniotic fluid, chorionic villi, placental tissue, skin fibroblasts, buccal epithelial cells, surgical specimens, and post-mortem tissues.

### 3.4. Molecular Findings and Proposed Mechanisms

The molecular findings and proposed pathogenic mechanisms identified across the included studies are summarized in Table 2. Considerable heterogeneity was observed regarding both the investigated phenotypes and the molecular techniques employed. Several studies identified discordant chromosomal abnormalities or tissue-specific chromosomal mosaicism, supporting postzygotic mitotic errors with unequal allocation of abnormal cell lines after embryonic splitting as a major mechanism underlying phenotypic discordance. Other reports described discordant copy number variants, whereas several studies demonstrated identical pathogenic variants in both twins despite markedly different clinical manifestations, suggesting variable expressivity rather than different genotypes. Epigenetic alterations emerged as another recurrent mechanism. Differential DNA methylation patterns, imprinting defects, and tissue-specific epigenetic mosaicism were reported in conditions such as congenital heart disease, Beckwith–Wiedemann syndrome, and Silver–Russell syndrome. In contrast, several studies using WES or WGS failed to identify discordant coding variants, supporting a role for epigenetic regulation, non-coding genomic variation, stochastic developmental events, or environmental influences. Placental factors also appeared to contribute substantially to phenotypic discordance. Multiple reports implicated twin-to-twin transfusion syndrome (TTTS), vascular anastomoses, blood chimerism, unequal placental sharing, and uteroplacental insufficiency as potential modifiers of phenotype, either independently or in combination with genetic and epigenetic mechanisms. Overall, the evidence suggests that discordant phenotypes in monozygotic twins rarely result from a single mechanism but instead reflect complex interactions among postzygotic genetic events, epigenetic modifications, placental physiology, and stochastic developmental processes.

## 4. Discussion

This systematic review summarizes the molecular evidence on discordant phenotypes in monochorionic twin pregnancies. We included 48 studies, published over five decades, with a total of 441 twin pairs. Most studies were case reports or small case series. However, the overall methodological quality was moderate to high. The included literature covered a wide range of phenotypes, but congenital heart disease, chromosomal abnormalities, sex chromosome mosaicism, imprinting disorders, and multisystem malformations were the most frequent conditions. The main finding of this review is that discordant phenotypes in monochorionic twins cannot be explained by a single biological mechanism. Instead, the available evidence supports a complex model. In this model, postzygotic genetic events, tissue-specific mosaicism, epigenetic changes, placental vascular factors, and stochastic developmental processes may interact. This is consistent with the biological nature of monochorionic pregnancy [61] These twins share a placenta and often share vascular connections, but they do not necessarily share the same developmental environment [13,62]. Postzygotic chromosomal events were among the clearest mechanisms identified [63]. Several reports described discordant aneuploidies, structural chromosomal rearrangements, or chromosomal mosaicism. Examples included trisomy 21, trisomy 17, trisomy 12p, terminal deletions, and sex chromosome abnormalities. These cases suggest that chromosomal errors may occur after fertilization and either before, during, or shortly after embryonic splitting. If abnormal cell lines are distributed unequally between the two embryos, one twin may become clinically affected while the other remains normal or less affected. In some cases, the abnormal cell line was present only in specific tissues. This finding is important because blood testing alone may underestimate or misrepresent the true distribution of mosaicism. Sex-discordant monochorionic twins were a particularly informative subgroup. Many of these cases involved 45,X/46,XY, 45,X/46,X,idic(Y), or related sex chromosome mosaicism. These reports show that phenotypic sex discordance in monochorionic twins does not always indicate dizygosity. It may result from postzygotic loss or rearrangement of sex chromosomes, followed by unequal tissue allocation of 45,X and Y-bearing cell lines. At the same time, recent reports of monochorionic dizygotic twins highlight another mechanism: fusion of two embryos with subsequent blood chimerism through placental vascular anastomoses. Therefore, in sex-discordant monochorionic twins, both zygosity testing and tissue-specific molecular analysis are essential [62,64,65]. Another important result is that identical pathogenic variants can be associated with different phenotypes. Several studies reported the same CNV or monogenic variant in both twins, but only one twin had severe clinical manifestations. This was observed in conditions such as Smith–Magenis syndrome, Alagille syndrome, Alström syndrome, *16p11.2* microdeletion, and *WT1*-related disease. These findings suggest that genotype alone may not determine phenotype. Variable expressivity, epigenetic regulation, placental factors, and stochastic developmental events may modify disease expression. This is highly relevant for prenatal counseling. The detection of the same variant in both twins does not necessarily imply identical prognosis. Epigenetic mechanisms emerged as a recurrent explanation, especially in imprinting disorders and congenital heart disease. Studies on Beckwith–Wiedemann syndrome, Silver–Russell syndrome, and cardiac malformations showed tissue-specific methylation defects or differentially methylated regions. In congenital heart disease, some studies found no pathogenic sequence differences but identified methylation changes in genes involved in cardiac development. These findings support the hypothesis that epigenetic dysregulation may contribute to discordant development, especially when WES, WGS, CMA, or karyotyping are uninformative. However, current evidence remains limited. Many studies analyzed blood, cord blood, or accessible tissues, which may not reflect the epigenetic status of the affected organ [66,67,68]. The placenta is another central factor. In monochorionic pregnancies, placental sharing is often unequal, and vascular anastomoses may create different hemodynamic conditions for each fetus. Several included studies implicated TTTS, TAPS, sFGR, blood chimerism, abnormal cord insertion, and uteroplacental insufficiency as possible contributors to phenotypic discordance [13]. This was especially evident in congenital heart disease and growth discordance. It is important to distinguish between these two categories when interpreting the reviewed cardiac findings: primary structural cardiac malformations (e.g., conotruncal defects, septal defects, tricuspid atresia, hypoplastic left heart) likely arise from early developmental disruption and are more directly relevant to the genetic and epigenetic mechanisms discussed in this review, whereas TTTS-related acquired valvar or outflow tract changes, such as the pulmonic stenosis or valvar dysplasia described in TTTS recipients [43], represent a functional consequence of chronic volume and pressure overload from abnormal intertwin blood flow rather than a primary structural malformation. Several of the included studies on discordant congenital heart disease captured both categories without consistently distinguishing between them [20,43], a limitation that should be considered when interpreting the pooled findings on cardiac discordance in this review. Thus, monochorionicity itself should be considered an active biological factor, not only a marker of monozygosity.

The methodological heterogeneity of the included studies is both a strength and a limitation. It reflects the evolution of molecular diagnostics from conventional karyotyping to multi-omics approaches. Older reports often relied on cytogenetics and blood-based analysis, whereas recent studies used CMA, WES, WGS, methylation profiling, and SNP-based zygosity testing. This improved diagnostic resolution. Notably, a single large retrospective cohort study [36] accounted for a disproportionate share of the pooled sample (193 of 441 twin pairs, 44%). Given this, the frequency estimates reported by that study, namely, discordant chromosomal aberrations in 9/119 karyotyped pairs and discordant CNVs in 3/55 pairs with a normal karyotype, should be given particular weight when interpreting the overall prevalence of genetic findings in this review, whereas conclusions drawn from the remaining, largely single-case-report literature should be interpreted with caution given their limited generalizability and likely bias toward unusual or clinically striking presentations. However, it also limits direct comparison between studies. In addition, many reports lacked systematic placental assessment, long-term follow-up, or analysis of multiple tissues. These gaps are important because mosaicism and epigenetic alterations may be highly tissue-specific. Furthermore, TTTS status was not systematically extracted as a discrete variable across studies reporting congenital heart disease, precluding a precise estimate of how many twin pairs with discordant CHD also had TTTS as a contributing factor. This review has clinical implications. First, discordance in monochorionic twins should prompt careful evaluation of both fetuses, even when one appears normal. Second, molecular testing should ideally include confirmation of zygosity, assessment of both twins, and analysis of more than one tissue when feasible. Third, normal results from blood-based WES or CMA do not exclude mosaicism, epigenetic disease, or placental mechanisms. Finally, genetic counseling should avoid deterministic interpretations. The prognosis may differ even when the same molecular alteration is identified in both twins. The findings of this review raise an important question: is it possible to determine, for a given discordant monochorionic twin pair, which single mechanism, postzygotic genetic event, epigenetic modification, placental vascular factor, or stochastic developmental variation, predominantly drives the observed phenotype? Nonetheless, several research directions could meaningfully advance the field’s ability to disentangle, or at least better characterize, their relative weight. Future studies should systematically apply multi-tissue and multi-omic profiling to both twins in a pair, rather than relying on a single accessible tissue such as peripheral or cord blood. As shown in several of the included studies, mosaicism and epigenetic alterations can be confined to specific tissues, and blood-based testing alone may substantially underestimate their contribution; paired analysis of blood, placenta, and, when feasible, an affected organ or fibroblast culture would allow more accurate attribution of discordance to a specific mechanism. Second, longitudinal follow-up would clarify if the relative contribution of genetic, epigenetic, and placental factors evolves over time, and whether early molecular findings predict long-term phenotypic trajectories. Third, large prospective cohorts that apply a uniform, protocolized battery of testing (karyotyping, chromosomal microarray, methylation profiling, and placental vascular assessment) to all monochorionic twin pairs are needed to overcome the substantial publication bias toward striking case reports evident in this review, where a single large cohort study accounted for a disproportionate share of the pooled sample. Taken together, these approaches would not necessarily yield a single definitive answer for every discordant pair, but they would substantially improve the field’s ability to estimate the relative and interacting contributions of the mechanisms identified in this review, with direct implications for prenatal counseling and clinical management. An interesting question raised by these findings is if twin pairs that develop clinically significant vascular imbalance, manifesting as TTTS or TAPS, are more likely to show genetic or epigenetic discordance than monochorionic pairs with balanced anastomoses. Although this review did not systematically stratify findings by TTTS/TAPS status, several included studies offer indirect support for a plausible link: unbalanced anastomoses facilitate blood chimerism and twin-to-twin cell trafficking, which was explicitly invoked as a contributing mechanism for shared blood mosaicism in multiple studies of sex chromosome discordance. This raises the possibility that blood-based molecular findings in TTTS/TAPS-affected pairs may, in some cases, reflect cell trafficking rather than the true distribution of a postzygotic event, potentially confounding zygosity and mosaicism assessments performed on blood alone. Future studies could address this directly by comparing the frequency and nature of genetic and epigenetic findings between monochorionic pairs with and without documented TTTS/TAPS, and by preferentially analyzing tissues less susceptible to blood exchange (e.g., skin fibroblasts, buccal mucosa) to distinguish true postzygotic discordance from apparent discordance driven by vascular cell trafficking.

## 5. Conclusions

In conclusion, phenotypic discordance in monochorionic twins is best understood as the result of interacting genetic, epigenetic, placental, and stochastic mechanisms. Monochorionic pregnancy offers a unique model to study human developmental variability. Future studies should combine detailed fetal phenotyping, placental pathology, multi-tissue molecular analysis, and long-term follow-up. This integrated approach will improve diagnosis, counseling, and understanding of developmental disease in monochorionic twins.

## Figures and Tables

**Table 1:** Methodological quality assessment of the included studies.

Author (Year)	Study Design	Quality Assessment Tool	Score
Gates (2023) [6]	Case report	JBI Case Report	8/8
Yuan (2023) [18]	Cohort study	NOS	8/9
Sousa Gomes (2022) [19]	Case report	JBI Case Reports	6/8
Imany-Shakibai (2021) [20]	Retrospective cohort	NOS	7/9
Cao (2021) [9]	Case report	JBI Case Reports	7/8
Cistaro (2021) [21]	Case report	JBI Case Reports	7/8
Kluge (2020) [22]	Case report	JBI Case Reports	7/8
Inamdar (2020) [11]	Case report	JBI Case Reports	6/8
Takahashi (2020) [23]	Case report	JBI Case Reports	8/8
Chen (2020) [10]	Case report + literature review	JBI Case Reports	7/8
Chang (2020) [24]	Case report	JBI Case Reports	7/8
Fontana (2020) [25]	Case report	JBI Case Reports	8/8
Shi (2020) [26]	Case report	JBI Case Reports	7/8
Homatter (2019) [27]	Retrospective cohort study	NOS	7/9
Lyu (2018) [28]	Case report/molecular case analysis	JBI Case Reports	7/8
Zhang (2018) [29]	Retrospective cohort study	NOS	7/9
Soares dos Santos Junior (2017) [30]	Case report	JBI Case Reports	6/8
Xu (2017) [31]	Case series	JBI Case Reports	8/10
Chang (2017) [32]	Case report	JBI Case Reports	8/8
Li (2017) [33]	Case report	JBI Case Reports	8/8
Hollander (2017) [34]	Case report	JBI Case Reports	7/8
Rock (2016) [35]	Case report	JBI Case Reports	8/8
Peng (2016) [36]	Retrospective cohort	NOS	7/9
Zhou (2015) [37]	Case report	JBI Case Reports	6/8
Izumi (2015) [38]	Case report	JBI Case Reports	8/8
Essaoui (2013) [39]	Case report	JBI Case Reports	8/8
Schrey (2013) [12]	Observational placental study	NOS	7/9
Yu (2012) [40]	Case report	JBI Case Report	7/8
Pauli (2012) [41]	Case report	JBI Case Report	8/8
Edlow (2011) [42]	Retrospective case series	JBI Case Series	7/10
Alrais (2011) [43]	Retrospective cohort	NOS	6/9
Castori (2011) [44]	Case report	JBI Case Report	7/8
Tierling (2011) [45]	Case report	JBI Case Report	8/8
Rijntjes-Jacobs (2010) [46]	Case report	JBI Case Report	6/8
Wuttikonsammakit (2010) [47]	Case report	JBI Case Report	6/8
Bohec (2010) [48]	Case report	JBI Case Report	8/8
Zech (2008) [49]	Case report	JBI Case Report	8/8
Yamazawa (2008) [50]	Case report	JBI Case Report	8/8
Robertson (2006) [51]	Case report	JBI Case Report	8/8
Rohrer (2004) [52]	Case report	JBI Case Report	7/8
Perry (2002) [53]	Retrospective cohort/database study	NOS	7/9
Shotelersuk (1999) [54]	Case report	JBI Case Report	7/8
Helderman-van den Enden (1999) [55]	Case report	JBI Case Report	8/8
Costa (1998) [56]	Case report	JBI Case Report	7/8
Reiss (1995) [57]	Neuroimaging observational study with embedded MZ twin comparison	JBI Case Report	7/8
Kaplowitz (1991) [58]	Case report	JBI Case Report	8/8
Rogers (1982) [59]	Case report	JBI Case Report	7/8
Karp (1975) [60]	Case report	JBI Case Report	6/8

Abbreviations: JBI, Joanna Briggs Institute Critical Appraisal Checklist; NOS, Newcastle–Ottawa Scale. Footnote: Methodological quality was assessed using the Joanna Briggs Institute (JBI) Critical Appraisal Checklists for case reports and case series, and the Newcastle–Ottawa Scale (NOS) for observational studies. JBI scores are reported as the number of criteria fulfilled out of the total applicable items (e.g., 7/8 or 9/10), whereas NOS scores range from 0 to 9, with higher scores indicating better methodological quality.

**Table 2:** Molecular findings and proposed mechanisms in monozygotic twin pairs with discordant phenotypes.

Author (Year)	Twin Pairs (n)	Chorionicity/Amnionicity	Monozygosity Confirmed	Confirmed How	Discordant Phenotype	Molecular Technique	Tissue Analyzed	Main Finding	Proposed Mechanism
Gates (2023) [6]	1	MCDA	Not stated	Genome sequencing performed but zygosity confirmation not explicitly reported	Congenital cranial dysinnervation disorder (Moebius syndrome-like phenotype)	Genome sequencing (GS)	Saliva	No pathogenic variants or discordant disease-causing variants identified	Stochastic event
Yuan (2023) [18]	3	MC-NR	Not stated	No zygosity-specific test reported (methylation/expression profiling only)	Congenital heart disease	WGBS, MeDIP-qPCR, qRT-PCR	Cord blood; fetal heart tissue for validation	379 DMRs mapped to 175 DMGs; *SPESP1* and *NOX5* were hypermethylated and showed lower expression in CHD samples	Epigenetic alteration/differential DNA methylation
Sousa Gomes (2022) [19]	1	MCDA	Not stated	Karyotyping only; zygosity presumed from monochorionicity, not molecularly confirmed	One twin normal; co-twin with horseshoe lung, right lung hypoplasia, tricuspid atresia/right ventricular hypoplasia, cleft lip/palate, pelvic right kidney	Conventional karyotyping	Amniotic fluid	Abnormal fetus had normal karyotype, 46,XX; autopsy confirmed multiple discordant malformations.	Postzygotic mutation, epigenetic change, placental/environmental factors, abnormal cord insertion/hemodynamic imbalance.
Imany-Shakibai (2021) [20]	23	MC-MA/DA	Not stated	Cohort-level testing; per-pair zygosity confirmation method not specified	Discordant congenital heart defects (18/23 pairs)	Karyotype, chromosomal microarray, FISH; exome sequencing in selected cases	Amniocytes/CVS and postnatal blood/serum	Discordant CHD was more frequent than concordant CHD; abnormal genetic testing was uncommon and not significantly different between groups	Environmental, epigenetic, placental/hemodynamic factors, especially TTTS-related flow imbalance
Cao (2021) [9]	1	MCDA	Yes	STR analysis	TTTS with one structurally normal twin and one twin with increased NT, VSD, hypoplastic left heart and Robertsonian translocation trisomy 21	Karyotyping, MLPA, STR analysis, array-CGH	Amniotic fluid/amniocytes; parental peripheral blood	MCDA monozygotic twins discordant for karyotype: twin A 46,XX; twin B 46,XX,+21,der(21;21)(q10;q10). STR confirmed monozygosity; extra chromosome 21 was paternal in origin.	Postzygotic Robertsonian trisomy 21 after twinning, or prezygotic paternal error followed by early trisomy rescue in one twin; unequal allocation/rescue of abnormal cell line.
Cistaro (2021) [21]	1	MCDA	Yes	Microsatellite analysis	Cri du Chat syndrome severity, neurodevelopmental impairment, and brain metabolism	Karyotype, FISH, microsatellite analysis, brain ^18F-FDG PET/CT	Peripheral blood lymphocytes	Both twins had mosaic del(5p) and monozygosity, but markedly different clinical severity and brain glucose metabolism	Different distribution of mosaic cell lines in brain tissue; possible compensatory hypermetabolism and stochastic developmental factors
Kluge (2020) [22]	1	MCDA	Yes	Zygosity testing (droplet digital PCR-based)	Discordant biochemical/clinical presentation of congenital adrenal hyperplasia	CYP21A2 PCR, Sanger sequencing, MLPA, droplet digital PCR, zygosity testing	Blood-derived DNA	Both twins carried maternal c.293-13C>G and mosaic paternal *CYP21A2* full-gene deletion/hybrid allele; monozygosity >99.999%	Postzygotic *CYP21A2* deletion before twinning, with differential distribution of mutant cell line; shared intrauterine androgen exposure likely contributed to virilization
Inamdar (2020) [11]	1	MCDA	Yes	SNP microarray (identical genotype)	Sex-discordant twins: phenotypic female with horseshoe kidney and phenotypic male without anatomic abnormalities	Karyotyping; FISH; SNP microarray	Peripheral blood lymphocytes	Both twins had 45,X/46,X,idic(Y) mosaicism with similar blood karyotypes; SNP microarray confirmed identical genotype and found a novel 99 kb 3p24.3 deletion involving *TBC1D5*.	Early postzygotic event with unstable isodicentric Y chromosome and mosaic 45,X cell line; different tissue distribution of mosaicism likely explains sex discordance.
Takahashi (2020) [23]	1	MCDA	Yes	SNP-array	Discordant external genitalia: one fetus phenotypically male; one phenotypically female with severe cystic hygroma/Turner-like features	CVS karyotyping; SNP-array; XY-FISH	Chorionic villi; umbilical cord DNA; internal genitalia, liver, heart, lung, adrenal gland, bone marrow, spine	SNP-array confirmed monozygosity. Both fetuses had 45,X/46,XY mosaicism, but 45,X cells were ~80.9% in the female fetus and ~18.1% in the male fetus.	Postzygotic sex chromosome loss before/around twinning with unequal allocation of 45,X and 46,XY cell lines; different mosaicism levels likely caused discordant genital phenotype.
Chen (2020) [10]	1	MC-NR	Yes	QF-PCR	Low-level mosaic trisomy 17 in Twin A at amniocentesis; Twin A had IUGR/TTTS and postnatal preaxial polydactyly, Twin B normal	Conventional karyotyping; QF-PCR; interphase FISH	Cultured amniocytes; parental blood; postnatal peripheral blood; buccal mucosal epithelial cells	Twin A: 47,XX,+17/46,XX mosaicism in amniocytes; Twin B: 46,XX. QF-PCR confirmed monozygosity and excluded UPD17. Both had normal blood karyotype and favorable development at 2 years 7 months.	Postzygotic chromosomal error with low-level mosaicism; possible confined/tissue-limited mosaicism; QF-PCR useful for zygosity and UPD exclusion.
Chang (2020) [24]	1	MCDA	No	No molecular testing performed	Peters anomaly (bilateral anterior segment dysgenesis with corneal opacity) affecting the donor twin only after TTTS	None	None	Prenatal ultrasound detected persistent bilateral fetal ocular opacity in the donor twin, initially suspected to be congenital cataract. Postnatal ophthalmologic examination confirmed Peters anomaly. No associated CNS or major systemic anomalies were identified.	Epigenetic and hemodynamic mechanisms related to TTTS (placental hypoperfusion of the donor twin) were proposed; a de novo genetic event could not be excluded because no molecular testing was performed.
Fontana (2020) [25]	1	MCDA	Not stated	XCI analysis performed but zygosity confirmation not explicitly reported	Beckwith–Wiedemann syndrome	Pyrosequencing, MS-MLPA, MLID profiling, WES, XCI analysis	Blood, buccal swab, placenta, umbilical cord	Both twins showed KCNQ1OT1:TSS-DMR LOM and MLID in blood; only the affected twin showed LOM in buccal swab and extraembryonic tissues	Mosaic epigenetic alteration with asymmetric tissue distribution
Shi (2020) [26]	1	MCDA	Yes (implied)	SNP array (shared inherited variant, zygosity not explicitly restated)	Cardiovascular abnormalities	Karyotyping, SNP array	Amniotic fluid	Both twins carried the same maternally inherited class II 1q21.1 microdeletion; only twin A had TGA, PVS, and VSD	Same CNV with variable expressivity; modifying factors not determined
Homatter (2019) [27]	38	MC-MA/DA	Not stated	Cohort-level karyotyping in selected cases only; zygosity method not specified	Discordant congenital malformations	Karyotyping in selected cases	NR	Discordant malformations occurred in 9.3% of MC twin pairs and in 86.4% of MC pairs with congenital anomalies	Genetic, epigenetic, and environmental mechanisms proposed
Lyu (2018) [28]	1	MCMA	Yes (implied)	Whole-genome sequencing (zygosity not explicitly restated)	DORV in one twin, normal heart in co-twin	WGS, RRBS methylome profiling, bisulfite sequencing, RT-qPCR	Whole blood DNA/RNA	No pathogenic genome differences; 1566 DMRs identified; *ZIC3* and *NR2F2* promoter hypermethylation associated with reduced expression in *DORV*	Epigenetic dysregulation, especially promoter hypermethylation of cardiac developmental genes
Zhang (2018) [29]	79	MCDA	Yes	SNP-based zygosity analysis	Cardiovascular anomalies	Karyotyping, CMA, SNP-based zygosity analysis	Amniotic fluid or fetal blood	Most cardiovascular anomalies occurred in only one fetus; discordant karyotypes or CNVs were identified in some MZ pairs	Genetic heterogeneity in MZ twins; possible unequal inner cell mass division, intrauterine factors, or epigenetic effects
Soares dos Santos Junior (2017) [30]	1	MCDA	Not stated	WES/Sanger validation; zygosity confirmation not explicitly reported	Bilateral congenital refluxing unobstructed megaureter in one twin; normal urinary tract in co-twin	WES, Sanger validation	Peripheral blood DNA	HNF1B SNV confirmed in proband, co-twin and mother; absent in 11 unrelated megaureter cases; no causal variant proven	Multifactorial CAKUT pathogenesis; possible genetic susceptibility plus epigenetic/intrauterine factors
Xu (2017) [31]	6	MC-NR	Yes	STR zygosity testing	CHD in one twin, normal heart in co-twin	STR zygosity testing, array-CGH/SNP array, WES, qPCR, Sanger validation	Peripheral blood DNA	No validated discordant CNVs, SNVs, or indels explaining CHD discordance	Non-CNV/exomic mechanisms: epigenetic differences, environmental factors, non-coding variants, placental/hemodynamic influences
Chang (2017) [32]	1	MCDA	Yes	STR analysis	Trisomy 21 (hydrops and cleft lip in one twin; normal co-twin)	Amniocentesis with karyotyping, STR analysis, placental and skin fibroblast karyotyping	Amniotic fluid, placental tissue, fetal skin fibroblasts, parental blood	Monozygosity confirmed by STR. One twin had non-mosaic trisomy 21, whereas the co-twin had a normal fetal karyotype but mosaic trisomy 21 confined to placental tissue.	Maternal meiosis II nondisjunction producing a trisomy 21 zygote followed by early post-twinning trisomy rescue (anaphase lag) in one embryo, resulting in a euploid fetus with mosaic trisomic placenta.
Li (2017) [33]	1	MCDA	Yes	SNP-based zygosity analysis	16p11.2 microdeletion phenotype, mainly cardiovascular anomalies and developmental delay	Karyotyping, CMA, SNP-based zygosity analysis, FISH	Amniotic fluid, parental blood, postnatal peripheral blood	Both twins carried the same de novo 244 kb 16p11.2 microdeletion including *SH2B1*; twin A had cardiac anomalies and developmental delay, while twin B had single umbilical artery only	Same CNV with variable expressivity; possible epigenetic, environmental, placental, or developmental modifiers
Hollander (2017) [34]	1	MCDA	Not stated	Clinically presumed identical twins; no molecular zygosity test reported	Variable Alström syndrome course, mainly discordant cardiomyopathy severity and obesity	ALMS1 sequencing in one twin; cardiomyopathy gene panel	NR	Pathogenic compound heterozygous *ALMS1* variants identified in one twin; diagnosis clinically inferred in the co-twin	Same monogenic disorder with variable expressivity; possible epigenetic or stochastic factors
Rock (2016) [35]	1	MCDA	Yes	SNP analysis	Holoprosencephaly, congenital heart defect and TTTS with discordant chromosome 7q deletion	CVS karyotype; amniotic fluid karyotype; chromosomal microarray; SNP analysis for monozygosity	Chorionic villi; amniotic fluid (both twins)	Twin B carried a terminal 7q34→qter deletion (including SHH), while Twin A had normal karyotype and microarray; SNP testing confirmed monozygosity.	Postzygotic mitotic error occurring after twinning, resulting in a discordant 7q deletion.
Peng (2016) [36]	193	MCDA	Yes	QF-PCR (in tested MCDA pairs)	Discordant congenital malformations, most commonly multiple anomalies and cardiac defects	Conventional karyotyping; chromosomal microarray analysis; QF-PCR for monozygosity	Amniotic fluid and/or fetal blood	Discordant chromosomal aberrations were found in 9/119 pairs; discordant CNVs in 3/55 pairs with normal karyotype. CMA confirmed monozygosity in all tested MCDA pairs. Large chromosomal abnormalities were more frequent than submicroscopic CNVs.	Postzygotic chromosomal errors, unequal allocation of abnormal cell lines after twinning, possible CNV differences; CNVs appeared to explain only a minority of discordant malformations.
Zhou (2015) [37]	1	MCDA	Yes	STR zygosity testing	Both twins with SMS deletion and FGR; one twin with VSD and pulmonary artery stenosis, co-twin without structural anomalies	Karyotype, SNP-array/CMA, STR zygosity testing, exome sequencing	Uncultured amniotic fluid DNA; parental blood	Both fetuses had de novo 3.7 Mb 17p11.2 deletion involving *RAI1*; no discordant CNV/exonic mutation explaining CHD discordance	Variable expressivity of SMS likely due to interacting genetic, epigenetic, placental and environmental factors; possible uteroplacental insufficiency contribution
Izumi (2015) [38]	1	MCDA	Yes (implied)	SNP array (zygosity not explicitly restated)	Alagille syndrome severity, mainly hepatic disease and growth restriction	JAG1 sequencing, karyotyping, FISH, SNP array	Peripheral blood	Both twins carried the same inherited *JAG1* missense variant; no discordant CNVs were detected	Same monogenic disorder with variable expressivity; prenatal hypoxia proposed as modifier
Essaoui (2013) [39]	1	MCDA	Yes	STR analysis	18q21.2qter deletion with severe IUGR and unilateral cleft lip/palate in one twin; normal phenotype in co-twin	Array-CGH, FISH, conventional karyotyping, microsatellite (STR) analysis	Amniotic fluid, fetal blood, placental/fetal tissues (skin, liver, tendon, muscle, lung, kidney, spleen, thymus, heart)	Array-CGH detected a terminal 18q21.2qter deletion in the affected twin, while prenatal amniocytes from the co-twin were normal. Postmortem analyses demonstrated low-level tissue mosaicism in both twins with opposite distribution of abnormal cells. Monozygosity was confirmed by STR analysis.	Early postzygotic structural chromosomal deletion before embryonic splitting, followed by unequal allocation of normal and deleted cell lines during twinning, producing tissue-specific mosaicism and discordant phenotypes.
Schrey (2013) [12]	15	MC-NR	Not stated	Placental molecular study; zygosity confirmation not explicitly reported	Discordant fetal growth (10 discordant, 5 concordant pairs)	qRT-PCR array, ELISA, immunohistochemistry, leptin promoter methylation analysis	Placenta	Smaller twins showed increased placental leptin, Flt1 and endoglin expression; leptin upregulation was associated with increased promoter methylation	Epigenetic regulation of placental gene expression
Yu (2012) [40]	1	MC-NR	Not stated	WT1 sequencing only; zygosity confirmation not explicitly reported	Incomplete Denys–Drash syndrome vs isolated steroid-resistant nephrotic syndrome	WT1 sequencing	Blood	Identical de novo heterozygous *WT1* R394W mutation identified in both twins	Same pathogenic variant with discordant phenotype; possible two-hit model for Wilms tumor
Pauli (2012) [41]	1	MCDA	Yes (implied)	SNP array (zygosity not explicitly restated)	Trisomy 12p syndrome	Karyotyping, FISH, array-CGH, microsatellite analysis, SNP array	Blood lymphocytes, buccal mucosa, hair root cells	Both twins showed trisomy 12p mosaicism in lymphocytes, but high-grade trisomy 12p mosaicism was present only in buccal and hair cells of the affected twin	Unequal tissue distribution of postzygotic chromosomal mosaicism; possible twin-to-twin blood transfusion
Edlow (2011) [42]	11	MCDA	Not stated	Karyotyping only; zygosity method not specified	Discordant markedly enlarged NT ≥ 3.5 mm; associated findings included chromosomal abnormalities, TRAP sequence, growth/fluid discordance, and structural anomalies	Conventional karyotyping by CVS/amniocentesis/PUBS when performed	Chorionic villi, amniotic fluid, fetal blood; placenta for pathology in most cases	Among 162 MCDA pairs, 11 had discordant enlarged NT. Chromosomal abnormalities were found in 3/8 karyotyped pairs; most cases had normal fetal anatomy/karyotype.	Postfertilization nondisjunction with mosaicism, confined placental mosaicism, monochorionic vascular complications, and twinning-related structural discordance.
AlRais (2011) [43]	29	MC-MA/DA	Not stated	Cohort-level echocardiography/karyotyping; zygosity method not specified	Discordant congenital heart disease, including laterality defects, ventricular hypoplasia, valvar dysplasia in TTTS recipients, conjoining, conotruncal defects, tricuspid atresia and VSD	Fetal echocardiography; karyotyping in selected cases	Fetal echocardiographic records; selected CVS/amniotic fluid samples	Among 356 referred twin pairs, 29 MC pairs had discordant CHD. Lesions included laterality defects, HLHS/ventricular hypoplasia, TTTS-related valvar lesions, conjoined hearts and other cardiac defects.	Differential epigenetic/environmental effects, abnormal laterality signaling during twinning, and placental vascular/hemodynamic factors, including TTTS-related acquired valvar disease.
Castori (2011) [44]	1	MCDA	Yes	Microsatellite/STR analysis	Phacomatosis cesioflammea/PPV in one twin; co-twin healthy except standard Mongolian spot	Microsatellite/STR analysis	Buccal cells	Monozygosity confirmed; one twin had extensive nevus flammeus with aberrant dermal melanocytosis, while the co-twin was clinically unaffected.	Early postzygotic mutation after embryo splitting; possible non-allelic twin spotting with asymmetric distribution of mutated cell lineages.
Tierling (2011) [45]	1	MC-NR	Yes (implied)	Microsatellite analysis	Beckwith–Wiedemann syndrome	DNA methylation analysis (bisulfite PCR/SNuPE), microsatellite analysis, DNA sequencing	Peripheral blood, skin fibroblasts, saliva, buccal swab	The affected twin showed *KvDMR1* hypomethylation in all tissues, whereas the unaffected twin showed normal methylation except in blood. No UPD or sequence variants of chromosome 11 were detected.	Tissue-specific postzygotic epigenetic alteration (*KvDMR1* loss of methylation); shared blood hypomethylation likely due to feto-fetal transfusion.
Rijntjes-Jacobs (2010) [46]	1	MCDA	Not stated	SNP-array performed but zygosity confirmation not explicitly reported	Discordance for severe Schimmelpenning–Feuerstein–Mims syndrome: affected twin with craniofacial nevus sebaceus, ocular abnormalities, cerebral abnormalities, cleft palate and neonatal death; co-twin normal	SNP-array; cytogenetic/infectious testing on amniotic fluid; placental dye injection	Peripheral blood lymphocytes; amniotic fluid; placenta; skin biopsy attempted but inadequate	One MCDA twin had severe SFM syndrome while the co-twin was unaffected. SNP-array on blood showed no CNVs.	Postzygotic mutation causing mosaicism, consistent with lethal autosomal dominant mosaic model.
Wuttikonsammakit (2010) [47]	1	MCDA	No	Authors explicitly stated zygosity could not be definitively confirmed (fibroblast culture not feasible)	One fetus with cystic hygroma, hydrops, ascites, pleural effusion and ambiguous sex; co-twin phenotypically normal	Conventional karyotyping	Fetal blood from both twins	Both fetuses showed the same blood karyotype, 46,XY/47,XYY mosaicism in a 2:1 proportion, despite marked phenotypic discordance.	Possible unequal allocation of abnormal blastomeres, postzygotic mitotic error, placental vascular effects, or blood chimerism through interfetal anastomoses.
Bohec (2010) [48]	1	MCDA	Yes	Microsatellite analysis	Discordant fetal sex; female fetus with Turner phenotype/hydrops and male fetus with 47,XYY features	Karyotyping; interphase FISH; microsatellite analysis	Amniocytes, renal cells, genital/testicular tissue	Amniocytes showed 45,X in the female twin and 47,XYY in the male twin; FISH showed variable 45,X/47,XYY mosaicism in both twins; monozygosity confirmed.	Postzygotic sex chromosome missegregation from an original 46,XY zygote, with unequal allocation of 45,X and 47,XYY cell lines after twinning.
Zech (2008) [49]	1	MCDA	Yes	Microsatellite (STR) analysis	Discordant sex (female/male) with TTTS	Karyotyping; microsatellite (STR) analysis	Amniocytes, lymphocytes, umbilical cord fibroblasts, placental cells	Monozygosity confirmed despite discordant sex. Cytogenetic findings supported origin from a 47,XXY conceptus with 46,XX/46,XY mosaicism.	Postzygotic loss of the X chromosome in one cell line and Y chromosome in another from an initial 47,XXY zygote, before or after twinning.
Yamazawa (2008) [50]	1	MCDA	Yes (implied)	Microsatellite analysis	Silver–Russell syndrome	Microsatellite analysis, H19-DMR methylation analysis (COBRA, bisulfite sequencing), X-inactivation analysis	Leukocyte DNA	H19-*DMR* hypomethylation was detected only in the affected twin, while the unaffected twin showed normal methylation	Postzygotic epigenetic alteration (loss of *H19-DMR* methylation)
Robertson (2006) [51]	1	MCDA	Yes	Zygosity analysis (microsatellite-based)	Melnick–Needles syndrome	FLNA sequencing, microsatellite analysis, mosaicism quantification	Blood leukocytes, skin fibroblasts	*FLNA* 3596C>T mutation was detected only in the affected twin; zygosity analysis confirmed monozygosity	Very early postzygotic *FLNA* mutation
Rohrer (2004) [52]	1	MCDA	Yes	Polymorphic marker analysis	Discordance for Ullrich–Turner syndrome; twin A with Turner phenotype and growth retardation, twin B phenotypically normal	Conventional karyotyping; FISH; polymorphic marker analysis	Peripheral lymphocytes, buccal smears, urinary cells	Both twins showed 45,X/46,XX mosaicism in blood, but buccal FISH showed twin A predominantly 45,X and twin B predominantly 46,XX; monozygosity confirmed.	Postzygotic X-chromosome loss after embryonic splitting; blood mosaicism likely due to vascular anastomoses/chimerism.
Perry (2002) [53]	5	MCDA	Yes	DNA-based zygosity testing	Congenital hypothyroidism due to thyroid dysgenesis	DNA-based zygosity testing; thyroid imaging	Leukocyte DNA	All monozygotic twins identified in the databases were discordant for thyroid dysgenesis	Postzygotic stochastic events, epigenetic mechanisms, or early somatic mutations
Shotelersuk (1999) [54]	1	MCDA	Yes	STR polymorphic marker analysis	Oral–facial–digital syndrome type 1	STR polymorphic marker analysis; X-inactivation assay; conventional karyotyping	Peripheral blood lymphocytes/lymphoblasts	Monozygosity confirmed; affected twin had *OFD1* phenotype, unaffected twin normal; X-inactivation patterns were similar.	Most likely postzygotic mutation in the affected twin; skewed X-inactivation considered unlikely.
Helderman-van den Enden (1999) [55]	1	MCDA	Yes (implied)	DNA fingerprinting	Fragile X syndrome severity and mental capacity	FMR1 Southern blot/PCR, DNA fingerprinting, FMRP immunostaining	Blood/lymphocytes	Twins had different CGG repeat patterns; the less affected twin showed premutation/full mutation mosaicism and *FMRP* expression in 26% of lymphocytes	Early postzygotic CGG-repeat instability/mosaicism
Costa (1998) [56]	2	MCDA	Yes	DNA zygosity testing with minisatellite probe	Sex-discordant MZ twins; female twins with Ullrich–Turner phenotype, male co-twins phenotypically male	Karyotyping; DNA zygosity testing with minisatellite probe	Peripheral blood lymphocytes; skin fibroblasts; gonadal tissue	Both pairs were monozygotic and showed sex-chromosome mosaicism. Females had 45,X cell lines in fibroblasts/gonadal tissue, while males showed 46,XY or 45,X/46,XY mosaicism depending on tissue.	Postzygotic Y-chromosome loss before, during, or after twinning; tissue-specific mosaicism/chimerism from twin-to-twin blood exchange.
Reiss (1995) [57]	1	MC-NR	Yes (implied)	DNA fingerprinting	Fragile X full mutation with discordant cognitive phenotype/intellectual disability	DNA fingerprinting; FMR1 Southern blot/CGG repeat and methylation analysis; MRI morphometry	Lymphocyte-derived DNA; brain MRI	Monozygotic twin girls had similar *FMR1* full mutation status but markedly discordant IQ; neuroanatomical differences involved cerebellar vermis, fourth/lateral ventricles, thalamus, amygdala and subcortical nuclei.	Regional differences in X-chromosome inactivation within the brain; possible undetected prenatal morbidity.
Kaplowitz (1991) [58]	1	MCDA	Yes (implied)	DNA marker analysis	Ullrich–Turner syndrome	Karyotyping, DNA marker analysis	Blood lymphocytes, skin fibroblasts	Both twins had 45,X/46,XX mosaicism in blood; affected twin had only 45,X cells in fibroblasts, while unaffected twin had only 46,XX cells	Early postzygotic X chromosome loss with separation of 45,X and 46,XX cell lineages; blood mosaicism likely due to placental anastomoses
Rogers (1982) [59]	1	MCDA	Yes	Blood group/HLA/serum protein analysis	Down syndrome/trisomy 21	Karyotyping, blood group/HLA/serum protein analysis	Blood lymphocytes, skin fibroblasts	Both twins showed trisomy 21 mosaicism in blood; fibroblasts were 47,XY,+21 in the affected twin and 46,XY in the unaffected twin	Postzygotic chromosomal event with blood mosaicism likely due to placental vascular anastomoses
Karp (1975) [60]	1	MC-NR	Yes	Blood/serum group studies (concordant markers)	Phenotypic sex discordance; female twin with gonadal dysgenesis/Turner-like phenotype, male twin phenotypically normal	Blood/serum group studies; karyotyping; buccal sex chromatin	Blood lymphocytes; buccal smears; gonads; fallopian tubes	Monozygosity supported by concordant genetic markers; female twin had mainly 45,X gonadal tissue and 45,X/46,XY mosaicism in fallopian tubes; male twin had normal 46,XY blood karyotype	Postzygotic chromosomal mosaicism; possible selection against 45,X cell lines in utero

Abbreviations: MC, monochorionic; MCDA, monochorionic diamniotic; MCMA, monochorionic monoamniotic; MC-NR, monochorionic with amnionicity not reported; CHD, congenital heart disease; TTTS, twin-to-twin transfusion syndrome; TAPS, twin anemia–polycythemia sequence; sFGR, selective fetal growth restriction; FGR, fetal growth restriction; NT, nuchal translucency; CVS, chorionic villus sampling; PUBS, percutaneous umbilical blood sampling; STR, short tandem repeat; SNP, single nucleotide polymorphism; CMA, chromosomal microarray analysis; array-CGH, array comparative genomic hybridization; CNV, copy number variation; FISH, fluorescence in situ hybridization; MLPA, multiplex ligation-dependent probe amplification; MS-MLPA, methylation-specific multiplex ligation-dependent probe amplification; QF-PCR, quantitative fluorescent polymerase chain reaction; WES, whole-exome sequencing; WGS, whole-genome sequencing; GS, genome sequencing; RRBS, reduced representation bisulfite sequencing; WGBS, whole-genome bisulfite sequencing; MeDIP-qPCR, methylated DNA immunoprecipitation quantitative polymerase chain reaction; qRT-PCR, quantitative reverse transcription polymerase chain reaction; RT-qPCR, reverse transcription quantitative polymerase chain reaction; DMR, differentially methylated region; DMG, differentially methylated gene; SNV, single nucleotide variant; MLID, multilocus imprinting disturbance; XCI, X-chromosome inactivation; PET/CT, positron emission tomography/computed tomography; VSD, ventricular septal defect; DORV, double outlet right ventricle; TGA, transposition of the great arteries; PVS, pulmonary valve stenosis; IUGR, intrauterine growth restriction; CAKUT, congenital anomalies of the kidney and urinary tract; SMS, Smith–Magenis syndrome.

## Data Availability

No new data were created or analyzed in this study. Data sharing is not applicable to this article.

## Supplementary Materials

The following supporting information can be downloaded at: https://www.mdpi.com/article/10.3390/genes17070832/s1, Table S1: Literature search strategy.

## Abbreviations

The following abbreviations are used in this manuscript:

array-CGH	Array comparative genomic hybridization
ART	Assisted reproductive technology
CAKUT	Congenital anomalies of the kidney and urinary tract
CHD	Congenital heart disease
CMA	Chromosomal microarray analysis
CNV	Copy number variation
COBRA	Combined bisulfite restriction analysis
CVS	Chorionic villus sampling
DMR	Differentially methylated region
DMG	Differentially methylated gene
DNA	Deoxyribonucleic acid
DORV	Double outlet right ventricle
FGR	Fetal growth restriction
FISH	Fluorescence in situ hybridization
GS	Genome sequencing
HCP	Healthcare professional
HLA	Human leukocyte antigen
HLHS	Hypoplastic left heart syndrome
IUGR	Intrauterine growth restriction
JBI	Joanna Briggs Institute
MC	Monochorionic
MCDA	Monochorionic diamniotic
MCMA	Monochorionic monoamniotic
MC-NR	Monochorionic with amnionicity not reported
MeDIP-qPCR	Methylated DNA immunoprecipitation quantitative polymerase chain reaction
MLID	Multilocus imprinting disturbance
MLPA	Multiplex ligation-dependent probe amplification
MS-MLPA	Methylation-specific multiplex ligation-dependent probe amplification
MZ	Monozygotic
NOS	Newcastle–Ottawa Scale
NR	Not reported
NT	Nuchal translucency
PCR	Polymerase chain reaction
PET/CT	Positron emission tomography/computed tomography
PICO	Population, Intervention/Exposure, Comparator, Outcomes
PRISMA	Preferred Reporting Items for Systematic Reviews and Meta-Analyses
PROSPERO	International Prospective Register of Systematic Reviews
PUBS	Percutaneous umbilical blood sampling
PVS	Pulmonary valve stenosis
QF-PCR	Quantitative fluorescent polymerase chain reaction
qRT-PCR	Quantitative reverse transcription polymerase chain reaction
RAI1	Retinoic acid induced 1
RRBS	Reduced representation bisulfite sequencing
RT-qPCR	Reverse transcription quantitative polymerase chain reaction
sFGR	Selective fetal growth restriction
SFM	Schimmelpenning–Feuerstein–Mims syndrome
SMS	Smith–Magenis syndrome
SNP	Single nucleotide polymorphism
SNV	Single nucleotide variant
STR	Short tandem repeat
TAPS	Twin anemia–polycythemia sequence
TGA	Transposition of the great arteries
TRAP	Twin reversed arterial perfusion
TTTS	Twin-to-twin transfusion syndrome
UPD	Uniparental disomy
VSD	Ventricular septal defect
WES	Whole-exome sequencing
WGBS	Whole-genome bisulfite sequencing
WGS	Whole-genome sequencing
XCI	X-chromosome inactivation
