# Supplementary material for: Genetic and Epigenetic Mechanisms Underlying Phenotypic Discordance in Monochorionic Monozygotic Twins: A Systematic Review
Source: Genes (Basel). 2026 Jul 21;17(7):832. doi: 10.3390/genes17070832 (PMC13411809; doi:10.3390/genes17070832)
Supplement: Supplementary file 1 [file genes-17-00832-s001.zip › genes-4432046-supplementary.pdf]

Supplementary Table S1. Literature search strategy

Database	Search strategy
**PubMed/MEDLINE**	((monochorionic[Title/Abstract] OR monozygotic[Title/Abstract] OR "identical twin*"[Title/Abstract]) AND (discordant[Title/Abstract] OR discordance[Title/Abstract]) AND (phenotype*[Title/Abstract] OR malformation*[Title/Abstract] OR anomal*[Title/Abstract] OR congenital[Title/Abstract] OR syndrome[Title/Abstract] OR defect*[Title/Abstract]) AND (mutation*[Title/Abstract] OR variant*[Title/Abstract] OR mosaic*[Title/Abstract] OR methylation[Title/Abstract] OR epigenetic*[Title/Abstract] OR karyotype[Title/Abstract] OR microarray[Title/Abstract] OR CMA[Title/Abstract] OR array-CGH[Title/Abstract] OR FISH[Title/Abstract] OR sequencing[Title/Abstract] OR exome[Title/Abstract] OR genome[Title/Abstract] OR CNV[Title/Abstract] OR "copy number"[Title/Abstract])) NOT (review[Publication Type])
**Embase**	('monochorionic twin pregnancy'/exp OR 'monozygotic twin'/exp OR monochorionic:ti,ab OR monozygotic:ti,ab OR 'identical twin*':ti,ab) AND (discordant:ti,ab OR discordance:ti,ab) AND (phenotype*:ti,ab OR malformation*:ti,ab OR anomal*:ti,ab OR congenital:ti,ab OR syndrome*:ti,ab OR defect*:ti,ab) AND (mutation*:ti,ab OR variant*:ti,ab OR mosaic*:ti,ab OR methylation:ti,ab OR epigenetic*:ti,ab OR karyotyp*:ti,ab OR microarray:ti,ab OR 'array cgh':ti,ab OR cma:ti,ab OR fish:ti,ab OR sequencing:ti,ab OR exome:ti,ab OR genome:ti,ab OR cnv:ti,ab OR 'copy number':ti,ab) NOT ('review'/it OR 'systematic review'/it OR 'meta analysis'/it)
**Scopus**	TITLE-ABS(monochorionic OR monozygotic OR "identical twin*") AND TITLE-ABS(discordant OR discordance) AND TITLE-ABS(phenotype* OR malformation* OR anomal* OR congenital OR syndrome* OR defect*) AND TITLE-ABS(mutation* OR variant* OR mosaic* OR methylation OR epigenetic* OR karyotyp* OR microarray OR CMA OR "array CGH" OR FISH OR sequencing OR exome OR genome OR CNV OR "copy number") AND NOT DOCTYPE(re)

No language or publication date restrictions were applied during the electronic database searches. Duplicate records were removed before screening. Reference lists of all included studies and relevant reviews were manually screened to identify additional eligible articles.
